# Eye Contact and Fear of Being Laughed at in a Gaze Discrimination Task

**DOI:** 10.3389/fpsyg.2017.01954

**Published:** 2017-11-08

**Authors:** Jorge Torres-Marín, Hugo Carretero-Dios, Alberto Acosta, Juan Lupiáñez

**Affiliations:** ^1^Mind, Brain and Behavior Research Center, Department of Experimental Psychology, University of Granada, Granada, Spain; ^2^Mind, Brain and Behavior Research Center, Department of Methodology of Behavioral Sciences, University of Granada, Granada, Spain

**Keywords:** gelotophobia, gaze discrimination, eye contact, emotional expression, emotional categorization

## Abstract

Current approaches conceptualize gelotophobia as a personality trait characterized by a disproportionate fear of being laughed at by others. Consistently with this perspective, gelotophobes are also described as neurotic and introverted and as having a paranoid tendency to anticipate derision and mockery situations. Although research on gelotophobia has significantly progressed over the past two decades, no evidence exists concerning the potential effects of gelotophobia in reaction to eye contact. Previous research has pointed to difficulties in discriminating gaze direction as the basis of possible misinterpretations of others’ intentions or mental states. The aim of the present research was to examine whether gelotophobia predisposition modulates the effects of eye contact (i.e., gaze discrimination) when processing faces portraying several emotional expressions. In two different experiments, participants performed an experimental gaze discrimination task in which they responded, as quickly and accurately as possible, to the eyes’ directions on faces displaying either a happy, angry, fear, neutral, or sad emotional expression. In particular, we expected trait-gelotophobia to modulate the eye contact effect, showing specific group differences in the happiness condition. The results of Study 1 (*N* = 40) indicated that gelotophobes made more errors than non-gelotophobes did in the gaze discrimination task. In contrast to our initial hypothesis, the happiness expression did not have any special role in the observed differences between individuals with high vs. low trait-gelotophobia. In Study 2 (*N* = 40), we replicated the pattern of data concerning gaze discrimination ability, even after controlling for individuals’ scores on social anxiety. Furthermore, in our second experiment, we found that gelotophobes did not exhibit any problem with identifying others’ emotions, or a general incorrect attribution of affective features, such as valence, intensity, or arousal. Therefore, this bias in processing gaze might be related to the global processes of social cognition. Further research is needed to explore how eye contact relates to the fear of being laughed at.

## Introduction

The term gelotophobia (*gelos* in Greek means *laughter*) refers to a personality trait characterized by a disproportionate fear of being laughed at by others (Ruch, 2009). Although this phenomenon was originally conceptualized as a psychopathological disorder (Titze, 2009), recent approaches have operationalized gelotophobia as an individual differences variable that also shows considerable variation in non-clinical samples (e.g., Ruch and Proyer, 2008). In this sense, those individuals scoring high in trait-gelotophobia —or gelotophobes— are described as neurotic and introverted and as having a paranoid tendency to anticipate derision and mockery situations (Ruch and Proyer, 2009). This misinterpretation of humor related-situations undermines their social interactions, as they are constantly expecting contempt and rejection from others individuals (Ruch et al., 2014a). Research on gelotophobia has gradually progressed over the past two decades (Ruch et al., 2008; Titze, 2009; Platt et al., 2012, 2013; Wu et al., 2016), leading to a theoretical framework of reference that includes major findings concerning both potential triggering causes and moderating factors (e.g., bullying, parental influences or sociocultural factors), as well as consequences (e.g., humourlessness or social withdrawal) linked to gelotophobia predisposition (Ruch, 2009; Ruch et al., 2014a). Nevertheless, it is important to note that still, the nature of the predisposing factors of gelotophobia remains unclear. Contrary to traditional assumptions about the appearance/origin of gelotophobia (Titze, 2009), the presence of the traumatic experiences of teasing during childhood and adolescence does not seem to be a differentiating or invariant aspect of the development of this humor-related trait (Ruch et al., 2010). Therefore, additional research areas such as, for example, perceptual biases toward relevant affective or social cues (e.g., gaze or eye contact) need to be explored. Indeed, it has been stressed that gelotophobia research needs to move toward a more comprehensive and accurate theoretical model (Ruch et al., 2014a).

Recent experimental research on the fear of being laughed at has advanced our knowledge about this phenomenon. For instance, Papousek et al. (2009) designed an experimental task in which participants were exposed to several emotionally contagious films displaying a positive (e.g., cheerfulness), negative (e.g., anxiety or sadness), or neutral mood, with the purpose of comparing gelotophobes’ and non-gelotophobes’ responses to the emotional states of other individuals. The results revealed that individuals with gelotophobia did not show a reduced emotional induction to positive emotions compared with non-gelotophobes; interestingly, however, they showed a higher degree of affective induction to negative emotions, that is, high scores of subjective anxiety or sadness after watching anxiety- or sadness-causing films, respectively. In line with the analysis of gelotophobes’ reactions concerning the affective states of others, Ruch et al. (2015) used the Facial Action Coding System to analyze the potential differences between gelotophobes and non-gelotophobes in joy and contempt responses to videos of laughter-eliciting emotions (e.g., amusement or relief). In particular, they found that gelotophobes exhibited reduced facial expressions of joy (i.e., joyful smiles) and more expressions of contempt when they were exposed to laughter-eliciting emotions. In a different study, Ruch et al. (2014b), by using interactions with virtual agents (i.e., human-like figures or avatars) investigated which features of avatar laughter were considered to be not genuine, threatening, or malicious among individuals who score high on gelotophobia. Their results indicated that, among other factors, a low or mid-level intensity of laughter, an inhibited facial expression, and exaggerated body movements that accompany the laughter may be perceived as more malicious among gelotophobes. In a further investigation, Papousek et al. (2014) developed a realistic and socially relevant context in which participants were interrupted while performing an arithmetic task. The nature of the interruption was manipulated in three experimental conditions: anger provocation together with laughter, anger provocation together with white noise, and no interruption. The cardiac responses of the participants were recorded during the experiment, with a specific reaction of individuals with gelotophobia emerging, that is, a heart rate deceleration in response to others’ laughter. According to these authors, this psychophysiological response would be associated with a higher inclination in gelotophobes to interpret laughter as a cue of social rejection. To sum up, gelotophobes, compared with non-gelotophobes, seem to exhibit differentiated emotional manifestations. They are more sensitive to the contagion of negative emotions, show fewer facial expressions related to positive affective states as joyful smiles, and exhibit specific physiological reactions to potential threatening laugher. However, despite the undeniable progress made in the understanding of gelotophobia, further experimental research and new research topics are necessary for deepening the role of the fear of being laughed at in gelotophobes’ processing of emotional information.

### Smiles, Eye Contact, and Gelotophobia

Numerous authors have discussed the variability of meanings ascribed to a smile as well as the implication of its degree of genuineness or authenticity (Ekman et al., 1990; Ekman, 2003; Johnston et al., 2010). Although a smile is generally labeled as an indicator of a positive affective state, this emotional expression may hide other motivations as to denote, for example, social hierarchy or to mask negative feelings (Niedenthal et al., 2010). Evidence exists that a smile perceived as false or as a non-enjoyment smile is evaluated more negatively and can even lead the perceiver to show less cooperation or trust in comparison with a genuine or enjoyment smile (Johnston et al., 2010). One of the main features related to the fear of being laughed at is the tendency to interpret benevolent or neutral humor-related situations as threatening or malicious (Titze, 2009). Consistent with these findings, gelotophobes also tend to perceive others’ smiles as less joyful and more scornful than non-gelotophobes do (Hofmann et al., 2015). This smile misattribution may disturb the adequate social integration of these individuals, thus constituting to the persistence of gelotophobia (Ruch et al., 2014a). Exploring all different cues that may support the recognition of smiles and that may facilitate correct access to the meaning of smiles, especially among individuals with a higher inclination to gelotophobia, is therefore important.

Previous research has indicated that gaze and eye contact play relevant roles in the processes of recognition and inference making with regard to the meanings of others’ smiles (Niedenthal et al., 2010). Indeed, gaze entails an essential information source for enhancing our understanding of other people’s intentions, facilitating adaptation to our environment and being particularly relevant during social interactions (Argyle and Cook, 1976; Baron-Cohen, 1994; Cañadas and Lupiáñez, 2012). In particular, according to the simulation of smiles (SIMS) model, eye contact could act as a trigger of an embodied simulation process by which an individual obtains information to identify and interpret smiles (Niedenthal et al., 2010). Another theoretical approach that has highlighted the importance of the gaze direction when individuals have to interpret the intentions or anticipate the actions of others is *theory of mind* (ToM). According to Baron-Cohen (1994, 1995), the capacity to make inferences about others’ states of mind, or the “mind reading” system, would consist of a set of modular components, among which would be an eye direction detector (EDD). This module would be involved in the identification of a gaze direction (e.g., direct or averted) and therefore in the subjective perception of being looked at (Cañadas and Lupiáñez, 2012).

It has already been proposed—as a tentative explanation—that an atypically developed ToM could be related to the underlying wrong attribution present in gelotophobes, which would lead them to interpret that people are not laughing with them but rather laughing at them during social interactions (Ruch et al., 2008). Given that gaze discrimination is associated with both access to adequate meanings of smiles as well as expectations about how someone is going to behave, providing useful information for interpreting their objectives or intentions (Hudson et al., 2009; Niedenthal et al., 2010; Hudson and Jellema, 2011), we decided to explore whether higher trait-gelotophobia could be associated with potential bias processing gaze discrimination or eye contact, especially when the looking face portrays a smile. In this sense, a fundamental difficulty in gaze discrimination might underlie interpretation biases, leading gelotophobes to wrongly interpret others’ smiles as malicious or false.

To test this point, we used a novel gaze discrimination task that Cañadas and Lupiáñez (2012) developed, with the objective of exploring the importance of social stimuli (i.e., eye contact) in spatial Stroop paradigms. These authors discovered that the identification of a gaze direction is quicker when a face is located to the left but looking to the right, or vice versa (incongruent condition), in comparison with when the face location and eyes’ direction match (congruent condition). This reverse congruency effect—classical results with non-social stimuli, such as arrows, show faster responses for congruency trials—was interpreted in terms of eye contact (e.g., responses are faster when a face located to the left looks to the right, i.e., at us). Moreover, further investigation revealed that the emotional charge of the facial expression modulated this eye contact effect (Jones, 2015). More specifically, Jones’s results indicated that the effect was stronger for happy and angry faces (approach-oriented emotions) than for neutral faces, and it was non-existent for fearful faces (avoidance-oriented emotions). According to Adams and Kleck (2003), approach-oriented emotions (i.e., happiness and anger) are those that are identified more quickly when the faces displaying these emotions feature direct gazes rather than averted gazes. On the contrary, avoidance-oriented emotions (i.e., fear and sadness) are those that are recognized more quickly when the faces feature averted gazes vs. direct gazes. In this sense, Jones (2015) pointed out that the differences in the observed eye contact effect could be due to the differential facilitation of the processing of each emotion depending on the eye contact condition (e.g., a direct gaze would facilitate the processing of anger or happiness, and an adverted gaze would facilitate the processing of fear or sadness).

## Experiment 1

The purpose of the first experiment was to explore the performance of individuals scoring high vs. low in trait-gelotophobia in a gaze direction discrimination task, which has been previously shown to index an eye contact effect. The emotional expression of the face whose gaze direction had to be discriminated was also manipulated to investigate whether emotion affected the observed eye contact effect as a function of the gelotophobia levels of the participants. We expected trait-gelotophobia to modulate the eye contact effect data, showing specific group differences in the happiness condition. It may be possible for gelotophobes to respond to happy faces in the same way they would respond to fear faces, that is, as an avoidance-oriented emotion. Additionally, to corroborate the adequacy of the “approach or avoidance oriented emotions” interpretation for the reverse congruency (i.e., eye contact) effect that Jones (2015) proposed, and to extend our understanding of the role of emotional expression in the modulation of gaze discrimination, we decided to incorporate faces portraying sadness into our experiment. In accordance with Jones (2015), we expected to replicate the previous results in happiness, anger, neutral, and fear stimuli; regarding sadness, we expected to find a pattern similar to fearful faces and different from angry or happy faces.

### Materials and Methods

#### Participants

From a total sample (*N* = 202) of undergraduate students, 40 (32 females, 8 males; age ranging from 17 to 34; *M* = 19.80, *SD* = 2.94) were selected on the basis of their either extremely high or extremely low scores in trait-gelotophobia, and they were assigned to one of two comparison groups (gelotophobes vs. non-gelotophobes). All participants took part in the experiment voluntarily and received course credits in exchange for their collaboration. They reported normal or corrected-to-normal vision and hearing.

In particular, the selection criterion was the participant’s score on the Spanish version of Geloph <15> (Ruch and Proyer, 2008; Carretero-Dios et al., 2010a). The gelotophobes group consisted of the 20 participants who had the highest trait-gelotophobia scores (18 females; 17–25 years; *M*_Geloph_ = 2.76; *SD*_Geloph_ = 0.35; Min_Geloph_ = 2.20; Max_Geloph_ = 3.27). According to a transcultural investigation (Proyer et al., 2009), gelotophobia scores can be set in the following categories: 1.0–2.0: no gelotophobia; 2.0–2.5: borderline fearful; 2.5–3.0: slight expression of gelotophobia; 3.0–3.5: marked expression of gelotophobia; and 3.5–4.0: extremely fearful of being laughed at. Therefore, of the 20 participants, five were classified as borderline fearful, seven as slight expression of gelotophobia, and eight as marked expression of gelotophobia. Meanwhile, the non-gelotophobes group was also made up of 20 participants but, in this case, with the lowest trait-gelotophobia scores. (14 females; 18–34 years; *M*_Geloph_ = 1.38; *SD*_Geloph_ = 0.25; Min_Geloph_ = 1.00; Max_Geloph_ = 1.80). These 20 participants were classified as having no gelotophobia. It should be noted that, in order to improve the comparability of the results, we ensured that both comparison groups had the same number of participants (*n* = 20).

The two reported experiments were conducted in accordance with the ethical standards of the 1964 Declaration of Helsinki, following an ethical protocol approved by the University of Granada. All participants participated voluntarily in the studies and provided signed written consent before participating in the experiment.

#### Instruments

The Spanish version of the Geloph <15> (Ruch and Proyer, 2008; Carretero-Dios et al., 2010a) consists of a self-report questionnaire that assesses trait-gelotophobia, A sample item is “when others laugh in my presence I get suspicious.” It includes 15 positively keyed items in a 4-point answer format ranging from 1 (*Strongly disagree*) to 4 (*Strongly agree*). Test reliability (Cronbach’s alpha) was α = 0.94 in the present sample.

#### Apparatus and Stimuli

In this experiment, stimuli presentation, timing, and data collection were controlled by using E-Prime 2.0 run on a standard personal computer (PC). Stimuli were presented on a 17″ screen running at a 1024 pixel × 768 pixel resolution. The stimulus material consisted of 40 different full-color photographs (dimensions = 152 pixels × 186 pixels or 5.5 cm × 6.0 cm) of four males and four females portraying either a happy, angry, fearful, neutral, or sad emotional expression. All faces were selected from the Karolinska Directed Emotional Faces (KDEF; Lundqvist et al., 1998). As the original photos featured faces that looked straight ahead, they were manipulated via Adobe Photoshop CS6 for the purpose of changing the gaze directions to the left and right sides. The main selection criteria for the faces were as follows: (a) The gaze was clearly visible while displaying each facial expression (Jones, 2015), and (b) the global hit rate accuracy scores of each individual displaying an emotional expression was higher than 0.49 (*M* = 0.66; *SD* = 0.10) (Goeleven et al., 2008).

#### Procedure

**FIGURE 1:**
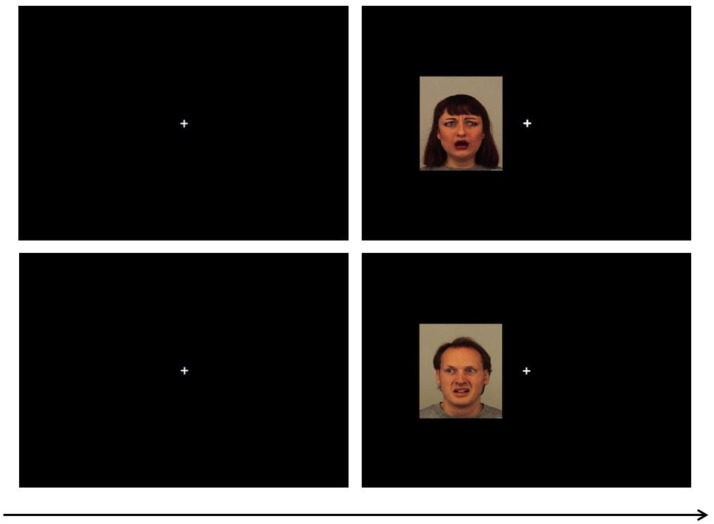
Procedure used in Experiments 1 and 2. The pictures located above illustrate a direct gaze trial (incongruent condition: a left-looking face located to the right), whereas the bottom pictures illustrate an averted gaze trial (congruent condition: a left-looking face located to the left).

We used a paradigm similar to that used in previous research (Cañadas and Lupiáñez, 2012; Jones, 2015). Participants performed an experimental task in which they had to discriminate the gaze directions (left or right) of faces that were presented to the left or to the right of fixation, by pressing, as quickly and accurately as possible, the corresponding key on the keyboard. Participants sat approximately 60 cm away from the monitor in a dimly illuminated testing room. Each trial began with the onset of a fixation point (a white cross: 0.5° × 0.5°) located in the center of a black computer screen for 500 ms. Then, a face portraying different emotional expressions was presented either to the left or to the right of the fixation point (approximately at 3.02° away from fixation to the inner edge of the face) and gazing either to the left or to the right (see **Figure 1**). Thus, considering that participants were in the middle, and following the interpretation by Cañadas and Lupiáñez (2012), the gaze direction could be either direct (e.g., a left-looking face presented to the right of fixation, i.e., potentially producing eye contact) or averted (e.g., a left-looking face presented to the left of fixation). Participants had to identify the face’s gaze direction by pressing, respectively, the “Z” or “M” key of the computer keyboard when the correct answer was left or right. Feedback on no-response or incorrect response trials was provided via a 220-Hz tone for 700 ms and a short text message. All possible combinations of stimuli, 8 (face identity) × 5 (emotional expression) × 2 (presentation side) × 2 (gaze direction), formed a total of 160 trials. Two blocks of trials with all combinations were presented for a total of 320 trials. Participants completed a practice block of 16 randomly selected trials to familiarize themselves with the task, followed by eight experimental subblocks of 40 randomly selected trials each, with a rest period between blocks. Participants could determine the duration of each rest period.

After performing the experimental task, participants had to fill out, again, the Geloph <15> (Carretero-Dios et al., 2010a) to ensure that they had been assigned to the appropriate groups and hence to enhance the validity of the obtained results.

#### Design

A 2 (gelotophobia: participants scoring high vs. low on Geloph <15>) × 5 (emotional expression: happiness, anger, fear, neutral, or sadness) × 2 (gaze direction: direct or averted) mixed design was used to analyze the data, with 32 observations per experimental condition. Response times (RTs) and error rates were used as dependent variables. The gelotophobia level was treated as a between-participant variable, and emotional expression and gaze direction as within-participant factors. A two-tailed significance level of *p* < 0.05 was used for all analyses.

### Results

#### Response Time

**FIGURE 2:**
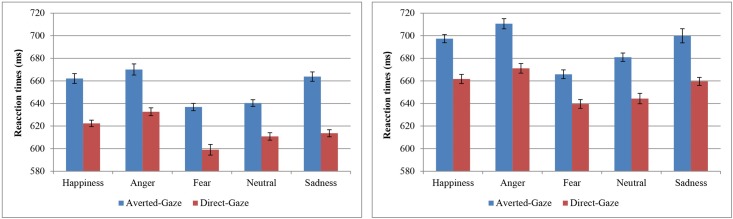
Responses time for gaze discrimination by emotional expression and gaze condition. The results obtained in the Experiment 1 are on the left and Experiment 2 on the right. Error bars represent standard error of the mean computed following Cousineau (2005) method.

Taking into account the procedure followed in the original study by Cañadas and Lupiáñez (2012), those trials with RTs shorter than 200 ms or slower than 1300 ms were eliminated from the RT analyses. Mean corrected RTs were submitted to a 2 (gelotophobia) x 5 (emotional expression) x 2 (gaze direction) mixed ANOVA. All response times (RTs) are measured and reported in ms. The results showed a main effect of emotional expression, *F*(4, 152) = 29.75, *p* <0.001, $\eta_{p}^{2}$ = 0.44, with the lowest reaction times being for fearful faces (*M* = 618; *SD* = 55.86) and the highest for angry faces (*M* = 651; *SD* = 60.72). Replicating Cañadas and Lupiáñez (2012), a main effect of gaze direction was also found, *F*(1,38) = 68.02, *p* < 0.001, $\eta_{p}^{2}$ = 0.64, with shorter RTs for direct gaze stimuli (*M* = 616; *SD* = 53.87) than for averted gaze stimuli (*M* = 655; *SD* = 65.40). Furthermore, as Jones (2015) showed, the interaction between emotional expression and gaze direction was significant, *F*(4,152) = 2.67, *p* = 0.035, $\eta_{p}^{2}$ = 0.07. However, in contrast to Jones’s (2015) conclusions regarding the interaction, paired *t*-tests showed that RTs were lower in the direct gaze than in the averted gaze condition for all emotional expressions [8.11 > *t*(39) > 5.54; all *ps* < 0.001, *d* = 0.47–0.79] (see **Figure 2**). Regarding trait-gelotophobia, no main effect of group was observed, *F*(1,38) = 0.15, *p* = 0.697, $\eta_{p}^{2}$ = 0.004. Furthermore, and importantly for our hypotheses, gelotophobia did not modulate any effect, especially the emotional expression × gaze direction interaction, *F*(4,152) = 0.40, *p* = 0.807, $\eta_{p}^{2}$ = 0.01.

#### Error Rates of Responses

**FIGURE 3:**
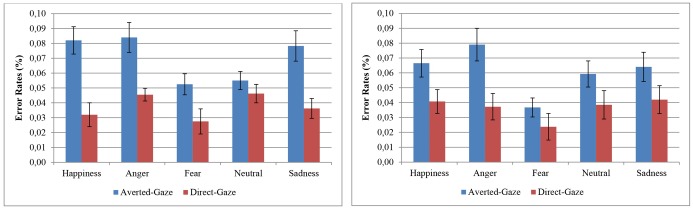
Error rates for gaze discrimination by emotional expression and gaze condition. The results obtained in the Experiment 1 are on the left and Experiment 2 on the right. Error bars represent standard error of the mean computed following Cousineau (2005) method.

In a similar pattern to the RT data, the obtained results showed a significant main effect of emotional expression, *F*(4,152) = 4.21, *p* = 0.003, $\eta_{p}^{2}$ = 0.10, with a higher error rate for responses to angry (*M* = 0.07; *SD* = 0.09) compared with fearful faces (*M* = 0.04; *SD* = 0.06). A main effect of gaze direction, *F*(1,38) = 10.17, *p* = 0.003, $\eta_{p}^{2}$ = 0.21, was also found, with lower error rates for direct gaze (*M* = 0.04; *SD* = 0.05) than for averted gaze stimuli (*M* = 0.07; *SD* = 0.10). Finally, as in the RT analysis, the interaction between emotional expression and gaze direction was significant, *F*(4,152) = 3.14, *p* = 0.016, $\eta_{p}^{2}$ = 0.08. To explore this interaction (see **Figure 3**), paired-samples t-tests were employed, and a greater error rate for averted gaze stimuli emerged for happiness, *t*(39) = 3.22, *p* = 0.003, *d* = 0.54; anger, *t*(39) = 3.17, *p* = 0.003, *d* = 0.29; and sadness, *t*(39) = 2.76, *p* = 0.011, *d* = 0.41. Furthermore, in spite of the results just bordered on a statistically significant value, *t*(39) = 1.98, *p* = 0.054, *d* = 0.29, a low effect size was observed for fearful faces in accordance with Cohen (1988) criteria. Lastly, no differences were found for neutral faces, *t*(39) = 0.84, *p* = 0.404, *d* = 0.12.

Concerning gelotophobia effects, our data revealed that this predisposition did not modulate the gaze discrimination error rate, *F*(1,38) = 2.32, *p* = 0.136, $\eta_{p}^{2}$ = 0.06, but an interaction close to statistical significance between gelotophobia and gaze direction appeared, *F*(1,38) = 3.56, *p* = 0.067, $\eta_{p}^{2}$ = 0.09. To explore this interaction, an independent analysis was performed on each gaze direction condition (direct vs. averted). Although our results failed to attain statistical significance at conventional levels, the Cohen values suggested that gelotophobes had higher error rates than non-gelotophobes, specially for averted gaze, *F*(1,38) = 2.91, *p* = 0.096, *d* = 0.50, compared to direct gaze, *F*(1,38) = 0.92, *p* = 0.343, *d* = 0.40.

Given that Ruch and Proyer (2008) derived empirical cut-off points for gelotophobia (≥2.50), and with the aim of avoiding potential limitations of our participant selection, we decided to repeat the above analyses but remove those participants classified as borderline fearful (*n* = 5) in the gelotofobia group. In addition, to balance the two comparison groups, we also removed the five participants of the no-gelotophobia group with the highest scores on the Geloph <15>. Thirty individuals composed our new test sample. Again, two comparison groups were created: 15 gelotophobes (14 females; 17–21 years; *M*_Geloph_ = 2.91; *SD*_Geloph_ = 0.24; Min_Geloph_ = 2.53; Max_Geloph_ = 3.27) and 15 non-gelotophobes (nine females; 18–34 years; *M*_Geloph_ = 1.26; *SD*_Geloph_ = 0.16; Min_Geloph_ = 1.00; Max_Geloph_ = 1.53). The RT analysis on the data from the more extremely selected sample did not change from the results with the whole sample. However, with regard to error rates, the new analysis indicated that gelotophobes had significantly higher error rates (*M* = 0.05; *SD* = 0.05) compared with non-gelotophobes (*M* = 0.02; *SD* = 0.01), *F*(1,28) = 8.59, *p* = 0.007, $\eta_{p}^{2}$ = 0.24. Additionally, the interaction between gelotophobia and gaze direction was also significant, *F*(1,28) = 4.99, *p* = 0.034, $\eta_{p}^{2}$ = 0.15. Again, an independent analysis was performed on each gaze direction condition (direct vs. averted). A between-participant effect emerged for the averted gaze condition, *F*(1,28) = 7.65, *p* = 0.010, *d* = 1.03, showing that gelotophobes had higher error rates (*M* = 0.08; *SD* = 0.08) compared with non-gelotophobes (*M* = 0.02; *SD* = 0.02). Along the same lines, a trend that approached significance and a low effect size, *F*(1,38) = 3.71, *p* = 0.064, *d* = 0.39, emerged for direct gaze stimuli, with higher error rates for gelotophobes (*M* = 0.03; *SD* = 0.03) than for non-gelotophobes (*M* = 0.02; *SD* = 0.02). Finally, and importantly for our hypotheses, the third-order interaction among gelotophobia, emotional expression, and gaze direction (see **Table 1**) did not reach statistical significance, *F*(4,112) = 1.33, *p* = 0.263, $\eta_{p}^{2}$ = 0.05.

**Table 1:** Means of reaction times (in ms) and error rates for gaze discrimination in Experiments 1 and 2 for each condition and gelotophobia group.

			High gelotophobia		Low gelotophobia	
				
	Emotional expression	Eye contact	RT	% Errors	RT	% Errors
Experiment 1	Happiness	Averted	669	0.10	655	0.07
		Direct	623	0.04	622	0.03
	Anger	Averted	673	0.11	668	0.06
		Direct	635	0.06	630	0.04
	Fear	Averted	644	0.08	630	0.02
		Direct	600	0.03	598	0.02
	Neutral	Averted	647	0.08	634	0.03
		Direct	616	0.06	606	0.04
	Sadness	Averted	666	0.12	662	0.04
		Direct	616	0.04	612	0.03
Experiment 2	Happiness	Averted	675	0.10	721	0.04
		Direct	641	0.06	682	0.03
	Anger	Averted	693	0.12	729	0.04
		Direct	643	0.05	699	0.02
	Fear	Averted	646	0.06	685	0.01
		Direct	613	0.04	666	0.01
	Neutral	Averted	655	0.09	707	0.03
		Direct	621	0.06	668	0.02
	Sadness	Averted	679	0.09	721	0.04
		Direct	640	0.06	679	0.02


### Discussion

As we expected, the results of the present experiment confirmed that gaze direction and emotion modulate reaction time in gaze discrimination. In line with Cañadas and Lupiáñez (2012), participants were faster and more accurate at identifying a gaze direction when the face was presented to the left but looking to the right (direct gaze) than the same face location but looking to the left (averted gaze). These data reinforce the eye contact interpretation of this reversed congruency effect and entail new evidence regarding its robustness. Importantly, our results indicated a similar pattern for RT and accuracy data in contrast to other authors who have reported that gaze direction does not modulate accuracy in a gaze-cueing paradigm (Prinzmetal et al., 2008). Furthermore, we found that emotional expression influenced our eye contact effect, but in a way that is inconsistent with the “approach and avoidance oriented emotions” interpretation by Jones (2015). In fact, the expression of sadness, which has been considered an avoidance-oriented emotion (Adams and Kleck, 2003), showed a pattern similar to that of approach-oriented emotions (e.g., happiness and anger). Similarly, emotional expression modulated the gaze direction effect in error rates as well. Participants showed lower error rates in identifying gaze directions with fearful faces compared with angry faces. In addition, more interestingly, direct gaze facilitated performance leading to higher accuracy, i.e., lower error rates for all emotional expressions with the exception of neutral faces. Therefore, although the “approach and avoidance oriented emotions” interpretation by Jones (2015) was not supported, the pattern of results supported the social nature of the reverse congruency effect observed, and therefore its interpretation in terms of eye contact (Cañadas and Lupiáñez, 2012). Eye contact is important in human communications (Doherty-Sneddon and Phelps, 2005), particularly in those interactions where emotional expression is present (Milders et al., 2011).

With respect to gelotophobia, and in relation to RT, we found no evidence for any modulation of gelotophobia in gaze discrimination. However, and interestingly, individuals with high trait-gelotophobia tend to make more errors when they have to discriminate gaze direction. The ability to detect correctly gaze direction is associated with the appropriate interpretation of others’ intentions (Baron-Cohen, 1994; Hudson and Jellema, 2011). Given that wrong attributions on the motivations and goals of other individuals could be considered one of the main components of gelotophobia (Ruch et al., 2008; Titze, 2009), this potential bias related to gaze identification could be a relevant finding to better understand the fear of being laughed at. Furthermore, the interaction between gelotophobia and gaze direction was significant, showing that the higher error rates observed in gelotophobes was larger in averted gaze trials than in direct gaze trials. Nevertheless, the independent analyses of the direct gaze condition also showed a low effect size for gelotophobia, so it seems necessary to explore this interaction further.

Finally, and in contrast to our hypothesis, happiness did not seem to have any special role in the observed differences between individuals with high and low trait-gelotophobia. Previous research has shown that gelotophobia may influence reactions to others’ affective states but not just those related to happiness (Papousek et al., 2009). However, inasmuch as we had to reduce our testing sample to adjust it to the reported cut-off points for gelotophobia (≥2.50) (Ruch and Proyer, 2008), we carried out an additional experiment to confirm the observed pattern of data, thus avoiding this potential limitation of the research. In addition, and importantly, to test whether our findings are specific to gelotophobia, in the next experiment, we controlled for social phobia as an alternative explanation of the observed effect of gelotophobia.

## Experiment 2

In Experiment 2, we tried to replicate the results observed in the preceding experiment, but controlling for the potential limitations highlighted above. The newly recruited participants for the high- and low-gelotophobia groups showed greater differences in their trait-gelotophobia scores. Then, we tested again whether individuals scoring high in trait-gelotophobia indeed have higher error rates in detecting gaze direction compared with individuals with lower trait-gelotophobia scores. Moreover, we explored the interaction between gelotophobia and gaze direction with the aim of confirming our previous finding that a larger gelotophobia predisposition could be associated with poorer performance, especially with averted gaze in comparison with direct gaze conditions. Finally, we were interested in analyzing the third-order interaction among gelotophobia, emotional expression, and gaze direction once again to corroborate that the happiness condition does not play any specific role in the eye contact effect that gelotophobes show.

Another main objective in gelotophobia research is to determine its differential features in relation to other disorders with similar symptomatology (e.g., social phobia) (Carretero-Dios et al., 2010b). Actually, previous research studies have reported that a high percentage of gelotophobes are also assessed as individuals with social phobia and/or Cluster A (i.e., schizoid, paranoid, or schizotypal) personality disorder (Weiss et al., 2012). Consequently, we included social phobia as a control variable to investigate whether the effects of gelotophobia could be explained on the basis of differences in social phobia.

In addition, we added a new experimental phase related to the identification of others’ emotional expressions. Although previous research indicated that gelotophobes did not have a general deficit in interpersonal emotion-related skills so as to categorize the emotions of others (Papousek et al., 2009), the goal of this second phase was to test whether eye contact conditions (direct vs. averted) modulate gelotophobes’ capacity to identify others’ emotional expressions. The manipulation of gaze direction in our preceding experiment seemed to be relevant, so we were interested in knowing whether gelotophobes would show a different pattern of emotion categorization depending on gaze conditions. Furthermore, all participants evaluated the intensity of the expressed emotion together with the valence and arousal of each face.

### Materials and Methods

#### Participants

Undergraduate students (*N* = 241) were screened using the Geloph <15>. The Sample included a total of 40 participants (32 females, 8 males; mean age of 21.18, *SD* = 6.34; range from 18 to 49) who were selected on the basis of their either extremely high or extremely low scores in trait-gelotophobia and assigned to one of the two comparison groups (gelotophobes and non-gelotophobes). As in Experiment 1, all participants reported normal or corrected-to-normal vision and hearing, and participants’ collaboration was in exchange for course credit. None of the participants had participated in Experiment 1.

The gelotophobes group was made up of 20 participants who had the highest trait-gelotophobia scores (16 females; 17–27 years; *M* = 20.00; *SD* = 3.06; *M*_Geloph_ = 2.93; *SD*_Geloph_ = 0.39; Min_Geloph_ = 2.53; Max_Geloph_ = 3.60). In contrast to Experiment 1, all participants in this study exceeded the cut-off point for gelotophobia (>2.50; see Ruch and Proyer, 2008). Thus, of these 20 participants with high trait-gelotophobia scores, none was classified as borderline fearful, 11 were classified as slight expression of gelotophobia, seven were classified as marked expression of gelotophobia, and two were classified as extremely fearful of being laughed at. Likewise, the non-gelotophobes group was made up of 20 participants whose scores were the lowest in the GELOPH <15> (16 females; 18–49 years; *M* = 22.35; *SD* = 8.39; *M*_Geloph_ = 1.24; *SD*_Geloph_ = 0.17; Min_Geloph_ = 1.00; Max_Geloph_ = 1.53). As in Experiment 1, these individuals were classified as having no gelotophobia.

#### Instruments

The Spanish version of the Geloph <15> was also used in this experiment with test reliability (Cronbach’s alpha) α = 0.96.

The Spanish version of the Social Interaction Anxiety Scale (SIAS; Mattick and Clarke, 1998; Olivares et al., 2001) consists of 20 items rated on a Likert-type scale ranging from 0 (*Not at all*) to 4 (*Totally*). A sample item is “I get nervous if I have to speak with someone in authority (teacher, boss, etc.).” In this study, the SIAS showed adequate good internal consistency (Cronbach’s alpha = 0.94).

#### Apparatus, Stimuli, and Procedure

The same procedure as in Experiment 1 was used in the first phase of this experiment. Additionally, a second experimental task was added in which participants had to identify the emotional expressions of faces with direct vs. averted gaze. For this new task, 160 photographs of 16 individuals, eight males and eight females, portraying either a happy, angry, fearful, neutral, or sad emotional expression, were also selected from the KDEF (Lundqvist et al., 1998). Stimuli were different from those used in the gaze discrimination task. Photographs did not have to be modified to recreate eye contact conditions. Each target face was presented for an unlimited time at the center of the monitor either with a direct gaze (i.e., the eyes looking straight ahead) or an averted gaze (i.e., the eyes looking left or right). Participants had to categorize the emotional expression by pressing the corresponding key on the keyboard (“1 = happiness,” “2 = anger,” “3 = fear,” “4 = neutral,” or “5 = sadness”). After each categorization, and while the picture remained visible, participants indicated their estimation of different affective dimensions—valence, intensity, and arousal—for that facial expression based on the Self-Assessment Manikin (SAM: Lang, 1980). Only one experimental block composed of 160 trials, 16 (faces) × 5 (emotion) × 2 (gaze direction), was created. Hence, we obtained 16 observations per gaze direction condition displaying each emotional expression. Trials were presented randomly for each participant. Finally, participants responded to gelotophobia and social phobia questionnaires, in that order.

#### Design

For the gaze discrimination task, the same design was used as in Experiment 1. For the analysis of the ratings in the emotional expression task, a similar design was used, 2 (gelotophobia: high trait-gelotophobia vs. low trait-gelotophobia) × 5 (emotional expression: happiness, anger, fear, neutral, or sadness) × 2 (gaze direction: direct or averted), with the following dependent variables (DVs): (a) *reaction time*; (b) *accuracy of responses* in the emotional categorization task; (c) *intensity* or magnitude of the emotion expressed (high vs. low); (d) *valence* or pleasantness of the faces displaying either emotional expression (positive vs. negative); and (e) the *arousal* or activation of these faces (active vs. calm). Again, gelotophobia predisposition was manipulated between participants, whereas the other variables were manipulated within participants. Furthermore, in all analyses, social phobia scores were introduced as a covariate to determine whether the specific effects are related to gelotophobia independently of social phobia. A two-tailed significance level of *p* < 0.05 was used for all analyses.

### Results

#### Gaze Direction Discrimination Task

*Response time data* showed, again, a main effect of emotional expression, *F*(4,152) = 30.16, *p* < 0.001, $\eta_{p}^{2}$ = 0.44, with the lowest reaction times for fearful faces (*M* = 653; *SD* = 64.71) and the highest for angry faces (*M* = 691; *SD* = 67.24). As in the previous experiment, a main effect of gaze direction was also found, *F*(1,38) = 41.18, *p* < 0.001, $\eta_{p}^{2}$ = 0.52, with participants having shorter RTs for direct gaze (*M* = 655; *SD* = 62.42) than averted gaze faces (*M* = 691; *SD* = 69.61). However, the interaction between emotional expression and gaze direction was not significant in this case, *F*(4,152) = 1.33, *p* = 0.26, $\eta_{p}^{2}$ = 0.03. Furthermore, there was a main effect of group, *F*(1,38) = 5.58, *p* = 0.023, $\eta_{p}^{2}$ = 0.13, with gelotophobes (*M* = 651; *SD* = 70.90) being faster compared with non-gelotophobes (*M* = 696; *SD* = 48.83). Nevertheless, this effect disappeared after controlling for social phobia scores, *F*(1,37) = 1.96, *p* = 0.170, $\eta_{p}^{2}$ = 0.05. As in the Experiment 1, the interaction between emotional expression and gaze direction was not modulated by gelotophobia, *F*(4,152) = 1.48, *p* = 0.210, $\eta_{p}^{2}$ = 0.04.

**FIGURE 4:**
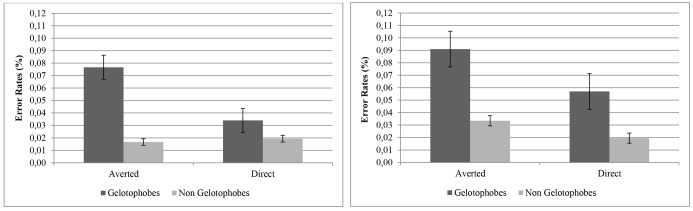
Error rates for gaze discrimination as a function of gaze condition and Gelotophobia group. The results from Experiment 1 are plotted on the left and those for Experiment 2 on the right. Error bars represent standard error of the mean computed following Cousineau (2005) method.

As in our previous experiment, the analysis of *error rate data* also showed a main effect of emotional expression, *F*(4,152) = 7.60, *p* < 0.001, $\eta_{p}^{2}$ = 0.17. Again, participants had the lowest error rate for fearful (*M* = 0.03; *SD* = 0.05) and the highest for angry faces (*M* = 0.06; *SD* = 0.07). However, the difference between direct gaze and averted gaze did not reach significance this time, *F*(1,38) = 2.70, *p* = 0.109, $\eta_{p}^{2}$ = 0.07, and neither was an interaction found between emotional expression and gaze direction, *F*(4,152) = 1.60, *p* = 0.179, $\eta_{p}^{2}$ = 0.04. Concerning gelotophobia, our results replicated the significant main effect of group, *F*(1,38) = 6.68, *p* = 0.014, $\eta_{p}^{2}$ = 0.15, with gelotophobes having higher error rates (*M* = 0.07; *SD* = 0.08) compared with non-gelotophobes (*M* = 0.03; *SD* = 0.03). Interestingly, this effect remained significant after controlling for individual social phobia scores, *F*(1,37) = 5.54, *p* = 0.024, $\eta_{p}^{2}$ = 0.13. Additionally, and in contrast to Experiment 1, the interaction between gelotophobia and gaze (see **Figure 4**) was not statistically significant, *F*(1,38) = 0.14, *p* = 0.708, $\eta_{p}^{2}$ = 0.004. Notwithstanding, a trend close to being significant and a medium effect size according to Cohen’ (1988) criteria, were found for the averted gaze condition, *F*(1,38) = 3.54, *p* = 0.067, *d* = 0.62, with gelotophobes having higher error rates (*M* = 0.09; *SD* = 0.13) compared with non-gelotophobes (*M* = 0.03; *SD* = 0.04), and it was significant for the direct gaze condition, *F*(1,38) = 8.65, *p* = 0.006, *d* = 0.79, with gelotophobia predispositions being associated, again, with higher error rates (*M* = 0.05; *SD* = 0.05) in comparison with lower gelotophobia (*M* = 0.02; *SD* = 0.02). Both effects remained after controlling for social phobia, *F*(1,37) = 3.59, *p* = 0.066, and *F*(1,37) = 4.80, *p* = 0.036, respectively.

#### Emotional Expression Categorization Task

The *analysis of RTs* showed a main effect of emotional expression, *F*(4,152) = 32.35, *p* < 0.001, $\eta_{p}^{2}$ = 0.46, with happiness faces identified significantly more quickly (*M* = 2137; *SD* = 614.52) than all other emotions were. Gaze direction and the interaction between emotional expression and gaze direction did not modulate any effect. It should be noted that RT was not limited in this phase. Furthermore, individuals with gelotophobia showed a tendency to respond more quickly when they identified others’ emotional expressions, *F*(1,38) = 5.87, *p* = 0.020; *d* = 0.77, $\eta_{p}^{2}$ = 0.13. Nevertheless, this effect disappeared after the inclusion of social phobia as a covariate, *F*(1,37) = 0.99, *p* = 0.327; $\eta_{p}^{2}$ = 0.03. On the other hand, the interaction between gelotophobia and emotional expression, *F*(4,152) = 2.02, *p* = 0.095, $\eta_{p}^{2}$ = 0.05, did not reach statistical significance. Finally, and interestingly with regard to our hypothesis, individuals with higher gelotophobia scores did not differ in their RTs due to gaze direction conditions, *F*(1,38) = 0.29, *p* = 0.865; $\eta_{p}^{2}$ = 0.001.

On the other hand, the *analysis of accuracy* data indicated a main effect of emotional expression, *F*(4,152) = 8.47, *p* < 0.001; $\eta_{p}^{2}$ = 0.18. The highest accuracy was observed for faces displaying happiness (*M* = 0.97; *SD* = 0.05) and the lowest for neutral faces (*M* = 0.86; *SD* = 0.15). On the contrary, no main effect of gaze direction, *F*(1,38) = 0.34, *p* = 0.566; $\eta_{p}^{2}$ = 0.01, was found, and the interaction between emotional expression and gaze direction did not reach statistical significance, *F*(4,152) = 1.47, *p* = 0.213, $\eta_{p}^{2}$ = 0.04. Importantly, the results showed that gelotophobia predisposition did not have any effect, *F*(1,38) = 0.40, *p* = 0.531; $\eta_{p}^{2}$ = 0.01, and did not modulate the effects of emotional expression, *F*(4,152) = 1.17, *p* = 0.328, $\eta_{p}^{2}$ = 0.18, or gaze, *F*(1,38) = 0.62, *p* = 0.805; $\eta_{p}^{2}$ = 0.002.

#### Emotional Expression Rating Task: Intensity, Valence, and Arousal

Emotional expression, *F*(4,152) = 15.80, *p* < 0.001, $\eta_{p}^{2}$ = 0.29, modulated the reported *intensity*. Particularly, the neutral expression had the lowest reported levels (*M* = 5.42; *SD* = 1.62) and the happiness expression the highest ones (*M* = 6.75; *SD* = 1.14). Gaze direction also modulated intensity, *F*(4,38) = 12.84, *p* < 0.001, $\eta_{p}^{2}$ = 0.25, with greater intensity levels reported for direct gaze (*M* = 6.20; *SD* = 1.03) than for averted gaze (*M* = 6.08; *SD* = 1.11). Furthermore, the interaction between emotional expression and gaze direction was also significant, *F*(4,152) = 3.95, *p* = 0.004, $\eta_{p}^{2}$ = 0.09. Paired t-tests showed that happy faces, *t*(39) = 5.30, *p* < 0.001, *d* = 0.34, with direct gazes (*M* = 6.94; *SD* = 1.09) were assessed with a greater level of intensity in comparison with happy faces with averted gazes (*M* = 6.54; *SD* = 1.23). A similar pattern was found for angry faces, *t*(39) = 1.98, *p* = 0.055, *d* = 0.14, with larger intensity reports for direct gaze (*M* = 6.41; *SD* = 1.19) than for averted gaze (*M* = 6.23; *SD* = 1.36). No differences were found for the other emotional expressions. The main effect of the gelotophobia group did not reach statistical significance, *F*(1,38) = 0.24, *p* = 0.628, $\eta_{p}^{2}$ = 0.01, although an interaction close to statistical significance between emotional expression and gelotophobia was observed, *F*(4,152) = 2.28, *p* = 0.063, $\eta_{p}^{2}$ = 0.06. Nevertheless, it completely disappeared after controlling for social phobia, *F*(4,148) = 0.93, *p* = 0.446, $\eta_{p}^{2}$ = 0.03.

Concerning valence and arousal, as expected, the *valence* ratings were modulated by emotional expression, *F*(4,152) = 172.83, *p* < 0.001, $\eta_{p}^{2}$ = 0.82. No evidence was found, however, that gaze direction modulated the valence ratings, *F*(1,38) = 0.21, *p* = 0.653, $\eta_{p}^{2}$ = 0.01. Interestingly, a significant interaction between emotional expression and gaze direction was also observed, *F*(4,152) = 3.37, *p* = 0.011, $\eta_{p}^{2}$ = 0.08. Paired *t*-tests, *t*(39) = 2.85, *p* = 0.007, *d* = 0.21, showed that happy faces with direct gazes (*M* = 7.27; *SD* = 0.98) were evaluated as more positive than happy faces with averted gazes (*M* = 7.06; *SD* = 1.02). In contrast, angry faces with direct gazes were evaluated as less positive (*M* = 3.05; *SD* = 1.02) than angry faces with averted gazes (*M* = 3.20; *SD* = 0.94), *t*(39) = -2.09, *p* = 0.044, *d* = 0.15. No differences for the other emotional expressions were found. On other hand, neither the main effect of gelotophobia, *F*(1,38) = 1.34, *p* = 0.255, $\eta_{p}^{2}$ = 0.03, nor its modulation over emotional expression, *F*(4,152) = 0.07, *p* = 0.992, $\eta_{p}^{2}$ = 0.002, or gaze direction, *F*(1,38) = 0.58, *p* = 0.451, $\eta_{p}^{2}$ = 0.02, reached statistical significance.

Finally, emotional expression also influenced the participants’ perceptions of *arousal*, *F*(4,152) = 48.64, *p* < 0.001, $\eta_{p}^{2}$ = 0.56, whereas gaze direction did not, *F*(1,38) = 0.31, *p* = 0.583, $\eta_{p}^{2}$ = 0.01. Nevertheless, the interaction between emotion and gaze was also significant, *F*(4,152) = 2.67, *p* = 0.034, $\eta_{p}^{2}$ = 0.07, and paired *t*-tests were used to explore this interaction. Differences in arousal were found for angry faces, *t*(39) = 2.53, *p* = 016, *d* = 0.20, with a greater arousal associated with direct gaze (*M* = 6.52; *SD* = 0.97) vs. averted gaze (*M* = 6.33; *SD* = 1.07). Finally, no main effect of group, *F*(1,38) = 0.42, *p* = 0.522, $\eta_{p}^{2}$ = 0.01, or interaction involving gelotophobia and emotional expression, *F*(4,152) = 0.10, *p* = 0.984, $\eta_{p}^{2}$ = 0.003, or gaze, *F*(1,38) = 0.48, *p* = 0.491, $\eta_{p}^{2}$ = 0.01, reached statistical significance for the arousal ratings. Those results concerning the abovementioned third interaction can be seen in **Table 2**.

**Table 2:** Means RTs and percentages of correct responses, and affective dimensions evaluations, for each condition and gelotophobia group, in the emotional categorization task of Experiment 2.

		High gelotophobia					Low gelotophobia				
			
Emotional expression	Eye contact	RT	% Correct	Intensity	Valence	Arousal	RT	% Correct	Intensity	Valence	Arousal
Happiness	Averted	2108	0.97	6.57	7.15	5.30	2236	0.97	6.52	6.97	5.64
	Direct	2113	0.96	7.05	7.37	5.43	2091	0.99	6.84	7.17	5.73
Anger	Averted	2975	0.88	6.17	3.28	6.36	3672	0.91	6.29	3.13	6.31
	Direct	2823	0.91	6.40	3.15	6.43	3590	0.93	6.43	2.94	6.63
Fear	Averted	3416	0.86	6.12	3.60	6.13	3876	0.88	6.20	3.32	6.18
	Direct	3369	0.84	6.12	3.48	5.90	3828	0.90	6.30	3.30	6.21
Neutral	Averted	2799	0.89	5.93	4.83	3.75	3575	0.84	4.99	4.81	3.93
	Direct	3042	0.87	5.70	5.02	3.76	3614	0.83	5.04	4.75	3.82
Sadness	Averted	3113	0.89	5.97	3.30	4.83	3638	0.93	6.04	3.24	5.01
	Direct	2905	0.87	6.16	3.31	4.82	3812	0.90	5.97	3.26	4.96


## General Discussion

In this study, we explored the modulation that a higher trait-gelotophobia produced in a task in which individuals were asked to discriminate the directions of the gazes of faces portraying different emotions. In particular, we were interested in examining the RTs and the error rates of gelotophobes to discriminate adequately the left-right direction of others’ eyes, as a function of whether they conformed direct vs. averted gaze conditions. To our knowledge, this is the first empirical study investigating the potential effects of gelotophobia in reaction to eye contact. In contrast to our initial hypothesis, and compared with non-gelotophobes, gelotophobes did not show a differential eye contact effect for happy faces. However, our results revealed a potential tendency among individuals with a greater degree of gelotophobia to make more error rates when identifying gaze direction. Interestingly, this potential bias in gaze discrimination is rather general, as it does not seem to be associated with a specific emotion or, according to our second experiment, the eye contact condition (direct or averted gaze). In fact, gelotophobes constantly exceeded—in terms of error rates—non-gelotophobes when they had to detect correctly the eyes’ directions of the different faces.

Detecting correctly gaze direction or eye contact is widely considered as a crucial factor in the communication of social intentions or desires (e.g., Argyle and Cook, 1976), to modulate social cognition processes as person categorization (e.g., Macrae et al., 2002) and also to obtain key elements concerning the mental states of others (e.g., Baron-Cohen, 1995). Traditional conceptualizations of gelotophobia have included a poorly developed social competence among its features (Titze, 2009). These limited social skills are characterized, for example, by a widespread fear of acting in a socially inadequate way (“maybe funny”), a feeling of insecurity, hypervigilance toward all possible contempt manifestations of social partners, and a general belief in the negative intentions of others (Platt et al., 2012; Ruch et al., 2014a).

In accordance with Baron-Cohen (1994), difficulties with discriminating others’ gaze direction could lead to wrong interpretations of others’ intentions or mental states. This is because individuals use the information provided by gaze in order to clarify ambiguous situations and, thus, to judge correctly intentions or acts of others (Phillips et al., 1992). Accordingly, a greater difficulty in knowing where other people are exactly looking at could be connected with the misattributions of others’ intentions that gelotophobes make during social interactions, as well as the incorrect access to the real meanings of some more complex emotional expressions. In this sense, given that gelotophobes seem to be less able to identify accurately the direction of other’s eyes (i.e., and therefore whether the attention is focused at a particular point or not), may contribute to perceive social interactions as ambiguous or, even, threatening. Furthermore, these results are in line with previous studies, which reported that gelotophobes show difficulties in adequately interpreting facially expressed communication (Ruch et al., 2014b).

In addition, it has been demonstrated that ambiguous eye contact conditions influence the subjective feeling of being observed (Senju and Hasegawa, 2006). Theoretical considerations and empirical data have supported the importance of studying eye contact and, more specifically, the feeling of being observed in relation to several disorders, such as social anxiety. For this reason, the goal of previous research was to determine the contextual cues that exacerbate the feeling of being looked at (Gamer et al., 2011). These authors found that a higher social phobia inclination would be associated only with a greater tendency to judge a “mutual gaze” in situations with a light social pressure (i.e., a second observer is present during the interaction), but not in one-to-one conditions. Given that our experimental setting recreated a one-to-one interaction, this may help with explaining why social anxiety cannot explain the bias revealed for gaze discrimination, i.e., why this bias rather seems to be specific to gelotophobia. Additionally, in our second experiment, we found that a higher gelotophobia predisposition could be related to faster responses regardless of eye contact conditions—direct or averted—in the gaze discrimination task. However, this effect disappeared after controlling for social phobia scores. These results could be due to the tendency of individuals with high social anxiety to be hypervigilant toward threatening social cues (Eysenck, 1992; Boll et al., 2016), such as eye contact, which is an indicator of the beginning of a social interaction.

Consistent with previous research (Cañadas and Lupiáñez, 2012; Jones, 2015), we replicated a reversed congruency effect. Furthermore, in general, the emotional expressions of faces modulate this effect: Although the interaction was not significant in Experiment 2, the same tendency was observed, and the combined analysis of the two experiments showed a significant interaction for both RT, *F*(4,316) = 2.48, *p* = 0.044, $\eta_{p}^{2}$ = 0.03, and error rates, *F*(4,316) = 3.30, *p* = 0.011, $\eta_{p}^{2}$ = 0.04. This is important, as it favors the interpretation of the reversed congruency effect in terms of eye contact. Nevertheless, it should be noted that in contrast to the pattern of results that Jones (2015) reported, the effect was also observed for the fearful expression; furthermore, sadness (theoretically, an avoidance-oriented emotion) showed a pattern similar to those of happiness and anger (approach-oriented emotion) in both experiments. For this reason, we cannot corroborate the “approach and avoidance oriented emotions” interpretation that Jones (2015) suggested. Thus, additional studies of our eye contact effect should look into other different frameworks used to explain the interaction between facial expression and gaze direction, as the *appraisal* theory (Sander et al., 2007). This theory focuses on the importance of the observer’s goals or intentions when interpreting or evaluating (appraisal process) the meaning of all of the external social clues (Sander et al., 2007; Milders et al., 2011). Perhaps, sadness could trigger avoidance motivation in others but also feelings of compassion or approach behavior to offer occasional help to the observer. Nevertheless, it is important to note the need for developing further research to elucidate the relationship between sadness and the reverse congruency effect data and, more generally, the role of emotional expression in this unusual effect. Furthermore, in this research, we incorporated the data of error rates in the gaze discrimination task. We observed a main effect of emotional expression, which was replicated in both experiments, with the highest error rates in faces expressing anger and the lowest in faces expressing fear. Importantly, the joint analysis of the error data in these two experiments revealed that this eye contact effect was stronger in faces displaying emotional expressions—with the exception of fearful faces—compared with neutral faces.

Concerning the emotional expression categorization task, we found that gelotophobes were faster when they had to categorize others’ emotional expressions, but as in the previous gaze discrimination task, this effect disappeared after controlling for social phobia scores. No accuracy differences between gelotophobes and non-gelotophobes in identifying others’ emotional expressions were found, with our results being consistent with the notion that gelotophobes do not present difficulties in the use of interpersonal emotion-related skills (Papousek et al., 2009). Interestingly, neither did we find any interaction among gelotophobia, emotional expression, and gaze direction for intensity, valence, or arousal. In sum, our results seem to indicate that eye contact conditions do not modulate the gelotophobes’ ratings of these affective dimensions.

Aside from gelotophobia effects, however, it should be noted that in this categorization task emotional expression modulated both RT and accuracy. More specifically, we found that happiness trials produced the fastest RTs and highest accuracy rates. Furthermore, the lowest accuracy rates were found in neutral faces. This data could be in line with previous studies reporting that emotional expressions, in comparison with neutral faces, facilitated processes such as face detectability (de Jong and Martens, 2007; Calvo and Nummenmaa, 2008; Milders et al., 2011). Along the same lines, we know that neutral faces contain affective keys more ambiguously than others’ emotional expressions. Indeed, authors as Zebrowitz et al. (2010) pointed out that neutral faces are often wrongly labeled as faces displaying anger in males or faces portraying surprise in females. In addition, a fewer number of neutral trials exist compared with emotional faces, which can lead to interpreting neutral faces as an emotional expression.

With respect to the affective dimensions measured, our results showed an interaction between emotion and gaze direction for intensity, valence, and arousal rates. More specifically, we found that faces displaying anger or happiness with direct gazes were evaluated as more intense than those with averted gazes. These results are consistent with other studies that have proposed that gaze direction modulates the recognition accuracy and perceived intensity of several emotions (Adams and Kleck, 2005). Consistent with intensity data, we obtained for valence an opposite pattern between anger and happiness. Whereas happy faces with direct gazes were rated as more positive than happy faces with averted gazes, angry faces with direct gazes were evaluated as more unpleasant than angry faces with averted gazes. Finally, differences in arousal were found for angry faces with a greater arousal associated with direct gaze than with averted gaze. The observed interaction between emotional expression and gaze direction fits with other empirical data suggesting that the processing of emotional expression and the processing of gaze pattern are interdependent (Ganel et al., 2005).

## Conclusion

The current results provide the first preliminary empirical evidence that gelotophobia is related to a potential bias in gaze discrimination. The effects of gelotophobia on error rates in discriminating gaze direction were replicated in two experiments. Furthermore, in the second experiment, the effect remained when controlling for social anxiety scores. Taking into account that gelotophobes, on the other hand, did not show any difference with non-gelotophobes in discriminating emotional expression, or intensity, arousal, or valence, our results could suggest that the gaze discrimination difficulties observed in high gelotophobes are not associated with problems with identifying others’ emotions or an incorrect attribution of affective features. These higher error rates in gaze direction accuracy might not be due to any limitation in processing affective information but rather might be related to global processes of social cognition. However, future research should clarify and continue exploring social cognition biases in gelotophobia to analyze the potential consequences of feeling being observed.

Several limitations of this research must be nevertheless pointed out. Firstly, due to the low prevalence of gelotophobes in non-clinical population, the sample sizes were relatively small. However, both the number of participants selected and the strategy adopted for recruiting them (i.e., construction of extreme groups) were in the line with previous research concerning gelotophobia (Papousek et al., 2014; Ruch et al., 2015). Lastly, it is important to indicate that some particular laughter (or humor)-related aspects were not included in these studies. Indeed, the use of pictures does not allow for the incorporation of key emotional components, such as sounds or movements. Therefore, it is possible that these stimuli may be insufficient to trigger some gelotophobes’ specific reactions and can help with explaining the absence of a specific effect on eye contact in the happiness condition. For this reason, future research should add other materials (e.g., films, virtual reality, etc.) with the aim of creating more realistic scenarios of emotional interactions where laughter is present, which will surely be a significant step forward for the main purpose of this research.

## Author Contributions

Conceived and designed the experiments: JT-M, HC-D, AA, and JL. Performed the experiments: JT-M. Analyzed the data: JT-M, HC-D, AA, and JL. Interpreted the data and drafted the manuscript: JT-M, HC-D, AA, and JL.

## Conflict of Interest Statement

The authors declare that the research was conducted in the absence of any commercial or financial relationships that could be construed as a potential conflict of interest.
